# Association Between the Morphology of Proximal Tibiofibular Joint and the Presence of Knee OA

**DOI:** 10.3389/fbioe.2020.610763

**Published:** 2020-12-18

**Authors:** Xin-Zheng Qi, Min Wang, Bo Zhang, Mao-Dan Nie, Xiao-Ying Ma, Hui-Zhi Wang, Xiao-Hong Wang, Cheng-Kung Cheng, Min Zhang

**Affiliations:** ^1^Beijing Advanced Innovation Centre for Biomedical Engineering, School of Biological Science and Medical Engineering, Beihang University, Beijing, China; ^2^Department of Orthopaedics, Xinqiao Hospital, Army Medical University (Third Military Medical University), Chongqing, China; ^3^Department of Orthopaedics, Beijing Chaoyang Hospital, Capital Medical University, Beijing, China; ^4^School of Biomedical Engineering, Shanghai Jiao Tong University, Shanghai, China; ^5^Beijing Medical Implant Engineering Research Center, Beijing Naton Technology Group Co. Ltd., Beijing, China

**Keywords:** knee osteoarthritis, proximal tibiofibular joint, knee morphology, 3D model, biomechanics

## Abstract

**Objective:** The aim of this study was to evaluate the association between the morphology of the proximal tibiofibular joint (PTFJ) and the presence of knee osteoarthritis (OA).

**Methods:** Twenty-eight OA subjects and 30 healthy subjects were enrolled in this study. A 3D model of the lower limb of each subject was constructed from CT scans and used to measure the characteristics of the PTFJ, including the shape of the articular facets, articular surface area, joint inclination, relative articular height, and joint declination. The association between the characteristics of the PTFJ and presence of knee OA was assessed using binomial logistic regression analysis.

**Results:** There was a significant difference between the OA and healthy groups in terms of the inclination (*p* = 0.028) and declination (*p* = 0.020) of the PTFJ and relative articular height (*p* = 0.011). A greater inclination angle (OR: 1.463, 95% CI: 1.124–1.582, *p* = 0.021), greater declination angle (OR: 1.832, 95% CI: 1.691–2.187, *p* = 0.009), and lower relative articular height (OR: 0.951, 95% CI: 0.826–0.992, *p* = 0.008) were found to be associated with an increased likelihood of knee OA being present.

**Conclusion:** The results of this study suggest that abnormal PTFJ morphology is associated with the presence of knee OA.

## Introduction

Knee osteoarthritis (OA) is a common degenerative disease affecting ~50% of individuals aged over 60, and is particularly prevalent in post-menopausal women suffering from osteoporosis (Pavelka et al., 2010). Symptoms tend to worsen over time and may require costly surgical treatment. Thus, understanding risk factors related to the onset of knee OA is beneficial for treating or preventing the disease at an early stage.

The proximal tibiofibular joint (PTFJ), referring to the synovial joint between the lateral condyle of the tibia and the head of the fibula, is located distally and laterally to the knee joint and plays a considerable role in maintaining stability of the lower limb (Barnett and Napier, 1952). The surface area of the PTFJ has been reported to vary from 0.17 cm^2^ (Ogden, 1974) to 3.26 cm^2^ (Espregueira-Mendes and da Silva, 2006). The joint morphology can be classified into 3 types according to the shape of the articular facets (Espregueira-Mendes and da Silva, 2006): plane type, trochoid type and double trochoid type. The PTFJ can also be classified as either horizontal type or oblique type according to the inclination angle between the fibular articular surface and the horizontal plane. Horizontal type joints have an angle of <20° (Espregueira-Mendes and da Silva, 2006), while oblique type joints have an angle of >20° (Ogden, 1974).

Proximal fibular osteotomy (PFO) has been demonstrated as an effective way of alleviating OA symptoms by resecting a section of bone on the proximal femur, which can alter the height of the medial knee joint space and improve the axial alignment of the lower extremity (Wang et al., 2017; Qin et al., 2018). Degeneration of the proximal tibiofibular joint has been shown to be associated with knee OA, and can result in knee pain on the lateral side (Oztuna et al., 2003). Thus, it is important to understand the relationship between PTFJ morphology and the presence of knee OA.

Using morphological measurements of the PTFJ, Zhao et al. (2018) demonstrated that the severity of knee OA was significantly correlated with the height of the fibula and shape of the PTFJ. Lu et al. (2017) also reported a significant association between the PTFJ shape and structural abnormalities of the knee joint in older adults. However, a major drawback to these studies is that the models only considered two-dimensional (2D) parameters to describe the anatomical characteristics of the three-dimensional (3D) PTFJ. Studies that considered the 3D morphology measurement appeared to be less affected by geometric factors (Crespo et al., 2014), and such methods were also beneficial for displaying the complex features of the joint (Chang et al., 2018). Three dimensional reconstruction of bone morphology has been effectively used on numerous other joints, including the femorotibial joint (Eckstein et al., 2001) and hip joint (Nakahara et al., 2014).

This study aimed to investigate whether the 3D morphology of the PTFJ is associated with the presence of knee OA. Three-dimensional models of healthy knees and OA knees were constructed from CT images and analyzed in terms of PTFJ type, tibiofibular articular surface area, joint angle, and the relative position of the proximal fibula to the tibia. It was hypothesized that the PTFJ morphology in OA knees was different from healthy knees, and that features of the PTFJ morphology could be associated with the presence of knee OA.

## Materials and Methods

### Participants

This study consisted of two subject groups: healthy group (*n* = 30 participants, 30 knees) and OA group (*n* = 28 patients, 28 knees). The participants in the healthy group were volunteers recruited through advertisements, while the OA group were recruited from a local orthopedic clinic. The inclusion criteria for the healthy subjects were having no history of knee diseases or functional abnormalities. For the OA group, invitation letters for participation were sent to all patients of the clinic who had previously been diagnosed with knee osteoarthritis K–L III and K–L IV using the Kellgren–Lawrence (K-L) grading system (Kellgren and Lawrence, 1957) on both compartments of the femorotibial joint between Jan 2016 and Feb 2016 (*n* = 40). Positive responses were received from 75% of patients (30/40). The inclusion criteria for the OA group were having no history of knee surgery, no rheumatoid arthritis or traumatic arthritis, and no underlying diseases, including hepatic and renal dysfunction. Of the patients that positively responded, 2 were excluded due to inadequate CT data, which would be used for the reconstruction of the PTFJ. Information on the participants is shown in Table 1. All protocols for this study were approved by the Beihang University Biological Science and Medical Engineering review board (No.: BM20200098) and written consent was obtained from all participants after being given detailed instructions on the protocols.

**Table 1:** Participant characteristics from healthy and OA groups.

		**Healthy group (*n* = 30 patients, 30 knees)**	**OA group (*n* = 28 patients, 28 knees)**
Gender	Male, *N* (%)/Female, *N* (%)	15 (50.0)/15 (50.0)	11 (39.2)/17 (60.7)
Side	Left, *N* (%)/Right, *N* (%)	15(50.0)/15 (50.0)	14 (50.0)/14 (50.0)
Age (years)		28.8 (2.1)	57.3 (5.9)
Height (cm)		164.2 (9.1)	160.2 (5.2)
Weight (kg)		66.2 (8.1)	60.2 (7.1)
BMI		20.3 (1.9)	21.2 (2.6)

*Values are represented as a mean (SD) unless otherwise indicated*.

### Construction of a Three-Dimensional Bone Model

Each subject was asked to lie in a supine position with knees fully extended so that the patella pointed straight up. Each subject was scanned with a CT scanner (SOMATOM Spirit, Siemens, Germany) from the distal femur to the ankle joint with a slice thickness of 0.5 mm. From the CT images, a 3D model of the PTFJ was constructed for each knee from both groups in Mimics 21.0 (Materialise, Leuven, Belgium). The method for reconstructing the 3D model was validated with cadaveric bone in a previous report by the authors (Zhang et al., 2020). The models in this current study consisted of a tibia and fibula, while soft tissues including ligaments, cartilage and menisci were excluded.

### Anatomical Measurement of PTFJ

**Figure 1:**
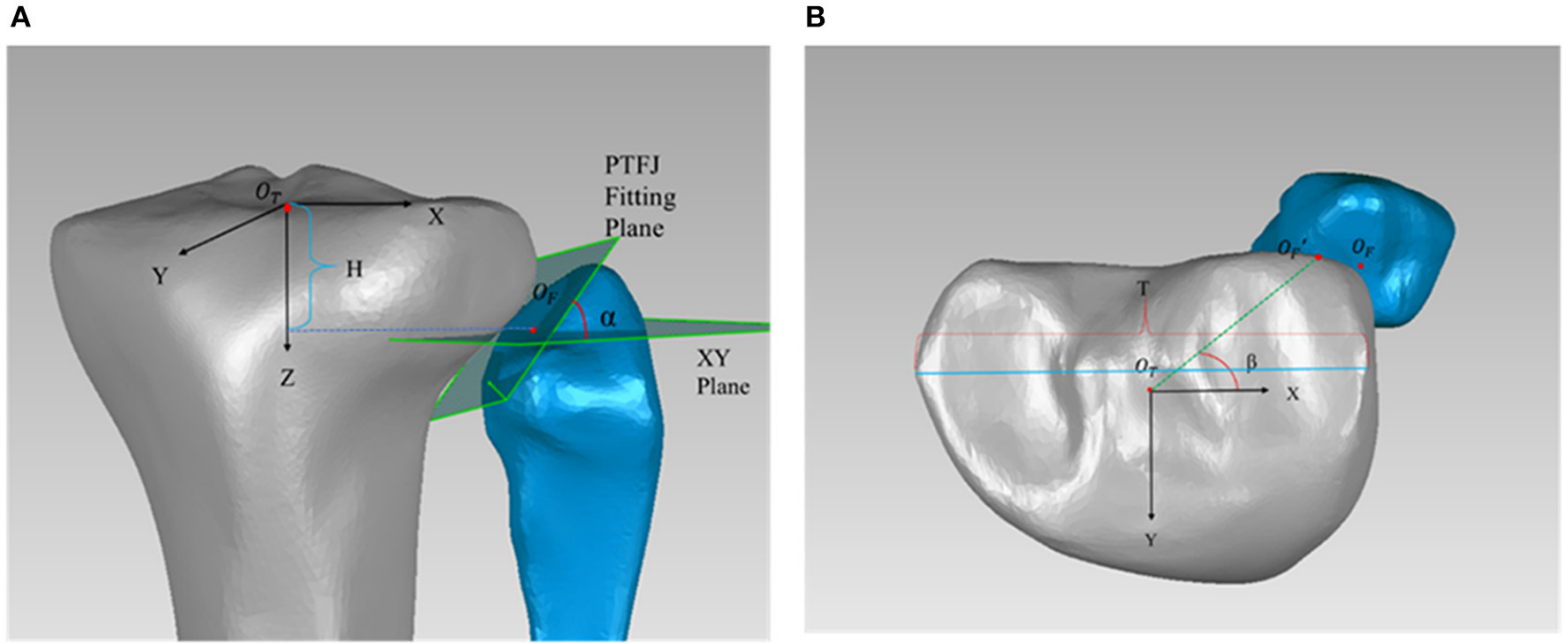
**(A)** Measurement of the articular height h (h = H/T), and inclination angle α; **(B)** Measurement of declination angle β. *O*_*T*_ is the center of tibial plateau and was used as the origin of the tibial coordinate system. The Z axis was determined by a line connecting the center of the tibial plateau to the center of the distal tibial articular surface, with the positive direction running from proximal to distal. The Y axis ran from posterior to anterior, and the X axis ran from medial to lateral. *O*_*F*_ was designated as the centroid of the articular surface on the fibular side. The inclination angle α was the angle between the fitting plane of the PTFJ and the XY plane. H was the distance between *O*_*F*_ and the XY plane. T was the largest medial-lateral width of the tibial plateau. The articular declination angle β was the angle between $O_{T}O_{F}^{\prime }$ and the X axis. $O_{F}^{\prime }$ was the projection of *O*_*F*_ in the XY plane.

The 3D models were imported into Geomagic Studio 19.0 (3D Systems, Morrisville, NC, USA) to evaluate the morphology. The PTFJ type, articular surface area, joint inclination and position of the PTFJ were recorded for comparison. This study classified each joint model according to the shape of the fibular articular facet: plane, trochoid and double trochoid (Espregueira-Mendes and da Silva, 2006). The articular surface area was determined as the fibular contact surface (Espregueira-Mendes and da Silva, 2006) and was calculated using the built-in surface area function in Geomagic Studio 19.0 (3D Systems, Morrisville, NC, USA) (accuracy: 0.01 mm^2^). The inclination angle of the PTFJ (α in Figure 1A) was calculated as the angle between the fibular articular surface and the XY plane. The inclination angle was measured three times for each model and the average value was taken as the value for the knee. Measurements from all knees in the same group (OA group and healthy group) were averaged and used as the value for that group.

The position of the PTFJ relative to the tibia was determined from the articular height and the articular declination angle (β in Figure 1B). To reduce the effect of individual variations, the articular height was expressed as a ratio using Equation (1), where h is the articular height, H is the distance from the centroid of the articular surface on the fibular side (***O***_***F***_) to the XY plane, and T is the largest medial-lateral width of the tibial plateau. This method was successfully used in the past for measuring PTFJ morphology (Zhao et al., 2018). The larger the value of h, the more distal the PTFJ is located relative to the tibial plateau. The articular declination angle (β) was calculated as the angle between $O_{T}O_{F}^{\prime }$ and the X axis (Equation 2). A larger value of β indicated a more posterior positioning of the PTFJ. Each measurement was recorded three times in Geomagic Studio 19.0, with the average being the value for that knee. These averaged measurements were then used to calculate the articular height (h) and articular declination (β). The intra-interclass correlation coefficient (ICC) was 0.927 for shape type, 0.821 for surface area, 0.908 for relative height, 0.823 for inclination, and 0.896 for declination, which suggested good reliability across all measures (Weir, 2005).

(1) $$\begin{array}{l} h=\frac{H}{T} \end{array}$$

(2) $$\begin{array}{l} \beta =arctan\left(\frac{Y}{X}\right) \end{array}$$

### Statistical Analysis

To minimize bias produced by similarities between the right and left knees of the same subject (Ohi et al., 2017), only one knee per subject was analyzed. In the OA group, if only one knee was diagnosed as having OA, only the affected side was selected for the study. If both knees in the same subject were diagnosed as having OA, the one with higher K-L grades was selected (Ohi et al., 2017). In the healthy group, the knee to be evaluated was randomly selected for each subject.

A univariate analysis was used to assess differences between the OA group and healthy group. The knees from both groups (healthy and OA) were summarized as a number (%) for categorical variables and as a mean [standard deviation (SD)] or median [interquartile range (IQR)] for continuous variables, as appropriate. The groups were compared using a Chi-square test for categorical variables and an independent *t*-test for continuous variables.

A binomial logistic regression analysis was performed in IBM SPSS 23.0 (IBM Corp., New York, USA) to determine whether the characteristics of the PTFJ were correlated with the presence of knee OA. The shape type, articular surface area, joint inclination angle, relative articular height, and joint declination angle were classified as independent variables, and the two groups (healthy group and OA group) were classified as dependent variables. Two conditions were studied using the regression models. In the first condition, only the independent variables outlined above were included to obtain the unadjusted association between the characteristics of the PTFJ and presence of knee OA. In the second condition, age, sex, and BMI were used as covariates to adjust the regression models to eliminate the influence of these factors on the results. Results were expressed as proportional odds ratios (ORs) with a 95% confidence interval (CI). A *p*-value of <0.05 was considered significant.

## Results

Table 2 summarizes the results obtained from the healthy group and OA group. The OA knees had a significantly larger joint inclination angle [39.64 (SD: 3.21)] than the healthy group [32.2 8 (SD: 5.92)] (*p* = 0.028). The relative position of the PTFJ with respect to the tibia in the OA group [0.25 (SD: 0.01)] was significantly more likely to be located proximally than the healthy group [0.32 (SD: 0.02)] (*p* = 0.012). The PTFJ in OA group [24.93 (SD: 2.14)] was significantly more likely to be located posteriorly than the healthy group [22.82 (SD: 1.99)] (*p* = 0.020). There were no significant differences between the healthy group and OA group in terms of the joint shape type [$\chi_{\left(3\right)}^{2}$ = 16.389, *p* = 0.452] and articular surface area of the PTFJ (*p* = 0.834).

**Table 2:** Characteristics of the PTFJ in the healthy group and OA group.

		**Healthy group (*n* = 30)**	**OA group (*n* = 28)**	***P***
Shape type	Plane type, *N* (%)	10 (33.3%)	12 (42.8%)	0.452
	Trochoid type, *N* (%)	16 (53.3%)	14 (50.0%)	
	Double trochoid type, *N* (%)	4 (13.3%)	2 (7.1%)	
Articular surface area of PTFJ (mm^2^)		432.13 (31.26)	420.19 (28.40)	0.834
Inclination of PTFJ (°)		32.28 (5.92)	39.64 (3.21)	0.028^*^
Relative articular height		0.30 (0.03)	0.25 (0.01)	0.011^*^
Declination of PTFJ (°)		22.82 (1.99)	24.93 (2.14)	0.020^*^

*Values are presented as a mean (SD) unless otherwise indicated*.

* *p < 0.05 was considered statistically significant. P-values from the chi-squared test for the categorical variables presented as N (%), and the independent t-test for the continuous variables presented as mean (SD)*.

The results of the binomial logistic regression model indicated that some characteristics of the PTFJ were statistically correlated with the presence of knee OA [$\chi_{\left(5\right)}^{2}$ = 21.293, *p* = 0.003 for the unadjusted model, $\chi_{\left(5\right)}^{2}$ = 20.817, *p* = 0.009 for the adjusted model]. The Hosmer and Lemeshow test *P*-value was 0.481 for the unadjusted model and 0.732 for the adjusted model, which indicated a high goodness of fit of the logistic model. The correlation between each joint characteristic and the presence of knee OA is shown in Table 3. The results in Table 3 indicate that a larger inclination angle was associated with an increased likelihood of exhibiting knee OA (OR: 1.317, 95% CI: 1.245–1.423, *p* = 0.019), even when adjusted for age, sex, and BMI (OR: 1.463, 95% CI: 1.124–1.582, *p* = 0.021). Similarly, a lower relative articular height was also correlated with the presence of knee OA (OR: 0.828, 95% CI: 0.681–0.952, *p* = 0.009), even in the adjusted model (OR: 0.951, 95% CI: 0.826–0.992, *p* = 0.008). A larger declination angle was significantly correlated with knee OA (unadjusted model: OR: 1.978, 95% CI: 1.562–2.759, *p* = 0.010; adjusted model: OR: 1.832, 95% CI: 1.691–2.187, *p* = 0.009), which indicated that the more posterior the head of the fibula relative to the lateral condyle of the tibia, the more likely the knee was to display signs of OA. The results in Table 3 also indicated that the shape type (OR: 1.000, CI: 0.997–1.002, *p* = 0.523) and articular surface area (OR: 1.891, CI: 0.892–1.923, *p* = 0.382) were not significantly correlated with the presence of knee OA. These findings for the adjusted model were consistent with the results for the unadjusted regression model.

**Table 3:** Logistic regression analysis on the association between characteristics of the PTFJ and knee OA (*n* = 58).

**Variables**	**Unadjusted model**			Adjusted model^*^		
	**OR**	**95% C.I**.	***p*-Value**	**OR**	**95% C.I**.	***p*-Value**
Shape type	1.000	(0.997, 1.009)	0.523	1.000	(0.997, 1.002)	0.721
Articular surface area	1.921	(0.781, 3.112)	0.782	1.891	(0.892, 1.923)	0.382
Inclination of PTFJ	1.317	(1.245, 1.423)	0.019	1.463	(1.124, 1.582)	0.021
Relative articular height	0.828	(0.681, 0.952)	0.009	0.951	(0.749, 0.998)	0.007
Declination of PTFJ	1.978	(1.562, 2.759)	0.010	1.832	(1.691, 2.187)	0.009

* *Adjustment for age, gender, BMI*.

## Discussion

This study aimed to investigate whether abnormal PTFJ morphology was associated with an increased presence of knee OA. The key findings of this study were: (1) The osteoarthritic knees had a significantly larger inclination angle for the PTFJ than those without OA; (2) The relative position of the PTFJ to the tibia in the OA group was significantly more likely to be located proximally and posteriorly compared to the healthy group; (3) The presence of knee OA was associated with a larger inclination and declination angle of the PTFJ and a lower relative articular height.

The surface area of the PTFJ has been reported to vary from 0.17 cm^2^ (Ogden, 1974) to 3.26 cm^2^ (Espregueira-Mendes and da Silva, 2006), due to differences in the location of measurement. As with Espregueira-Mendes' study, this current study used the fibular articular surface area as the surface area for measurement. The average surface area of the PTFJ in our study was 4.23 cm^2^ for the healthy group and 4.20 cm^2^ for the OA group, which were slightly larger than the 3.26 cm^2^ area reported by Espregueira-Mendes, although the same position was measured. The variation could be explained by the measurement methods used. Espregueira-Mendes assumed the articular surface to be approximately elliptical and the area was calculated using the minimal and the maximal diameters, whereas in our study the area of the whole curved fibular articular surface was calculated using a graphics function in Geomagic Studio 19.0 (3D Systems, Morrisville, NC, USA) (accuracy: 0.01 mm^2^). However, the discrepancies between the results were minor, which supported the reliability of our study.

The OA knees had a significantly larger inclination angle of the PTFJ and lower relative articular height than those without OA. These findings were consistent with a study by Zhao et al. (2018), in which subjects with OA were more likely than the healthy group to have a PTFJ inclination angle >20° (84.4 and 80.8%, respectively), and the fibula in OA knees was more likely to be located proximally than in healthy knees. Also, a novel finding of this study was that the PTFJ was located more posteriorly in OA group than healthy group.

**Figure 2:**
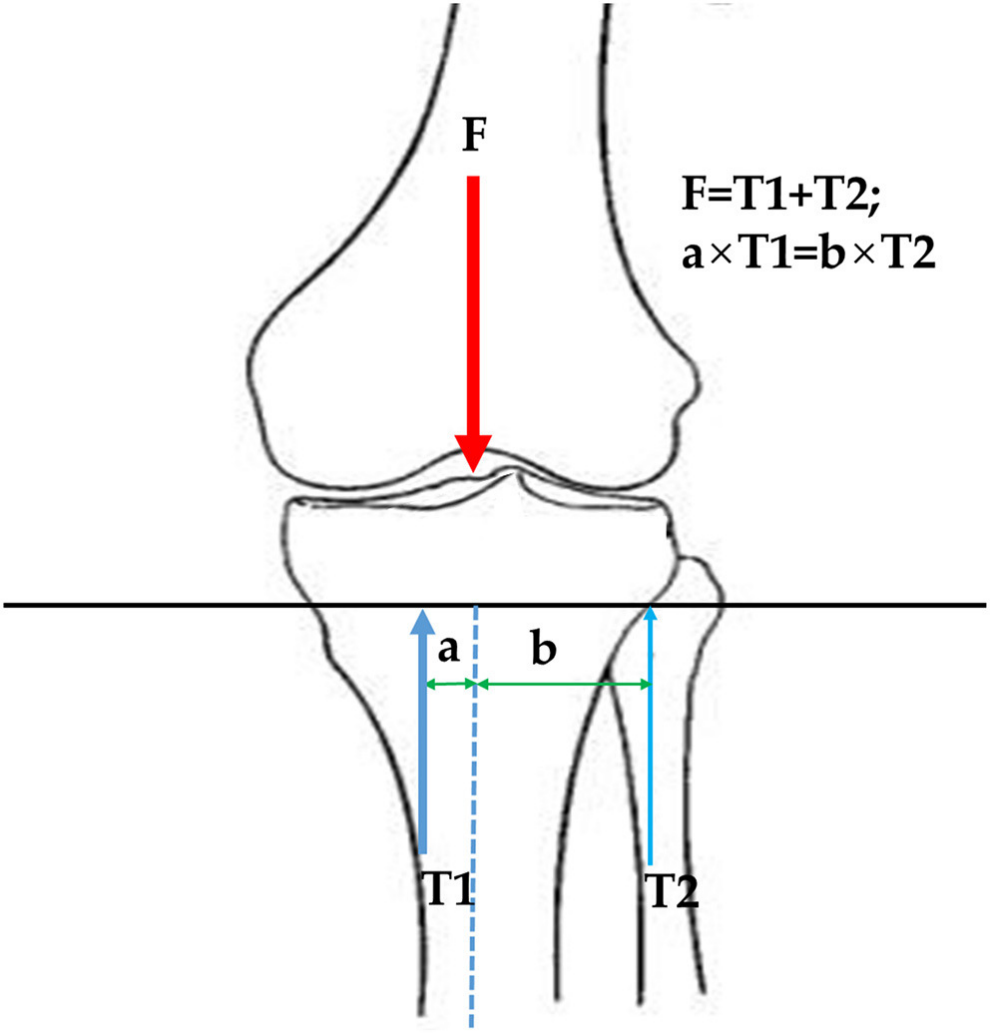
Influence of PTFJ inclination angle on tibial force. F is loading from the femur; T1 is the support force from the tibia; T2 is the support force from the fibula; a is the moment arm of tibial force; b is the moment arm of fibular force.

The logistic regression study revealed that the presence of knee OA was associated with the morphology and position of the PTFJ with regards to the inclination angle and relative articular height in the adjusted model. This finding was consistent with results reported by Zhao et al. (2018), in which patients with a large inclination angle (oblique type PTFJ) were more likely to display knee OA than those with small inclination angle (horizontal type PTFJ), and the subjects with a greater relative articular height were less likely to display knee OA than those with a lower relative articular height. By utilizing 3D measurements of PTFJ morphology, this study also found that the presence of knee OA was correlated with the declination angle of PTFJ. This may be explained by the fibula acting as a support mechanism for the tibia and bearing a portion of the load from the femur, ranging from 6.4% (Lambert, 1971) to 16.7% (Takebe et al., 1984). An increase in the inclination angle of the PTFJ may result in a reduction in the load borne by the fibula and thus an increased force borne by the tibia. Additionally, the medial compartment of the tibia suffers greater loading than the lateral compartment (Dayal et al., 2005), which may subsequently increase the risk of damage to the tibial cartilage and make the joint more likely to develop osteoarthritis (Wise et al., 2012). When the PTFJ is located more proximally (lower relative articular height), the moment arm of the fibula (b in Figure 2) is increased as the mediolateral region of a tibial platform is larger than the anteroposterior region (Zhang et al., 2019). This may lead to a reduced force on the fibula (T2 in Figure 2) and increased force on the tibia (T1 in Figure 2). The unbalanced loading may similarly increase the risk of developing knee OA (Figure 2). When the PTFJ is positioned posteriorly relative to the tibia, the load borne by the fibula is also reduced due to the reduced moment arm, leading to increased loading on the tibia, which again can increase the risk of developing knee OA.

This study also revealed that the shape type of the PTFJ and articular surface area were not associated with the presence of knee OA. Different shape types and variations in the articular surface area possibly did not change the relative position of the fibula relative to the tibia, and may not lead to a change in force on the tibia. Therefore, these parameters were not associated with the presence of knee OA. However, Lu et al. (2017) reported that an irregular PTFJ shape may increase the risk of developing OA-related cartilage defects, bone marrow lesions and osteophytes in the lateral compartments. The difference may be explained by Lu's study considering cartilage defects, bone marrow lesions and osteophytes as the variables, which would be expected to cause a highly irregular contact surface. These factors were not incorporated into this current study as the variable of interest was the presence of knee OA. Similarly, Chang et al. (2020) reported that increasing the contact area of the PTFJ may increase the risk of developing knee OA. In their study, the articular contact surface area was calculated according to the projection areas on the horizontal, sagittal and coronal planes, which were measured through MR images of the PTFJ joint. In our study, the surface area of the whole curved fibular articular surface was calculated using a graphics function in Geomagic Studio 19.0 (3D Systems, Morrisville, NC, USA) (accuracy: 0.01 mm^2^). The different measurement methods for the surface area and differences in the observation period may explain the inconsistent findings between Chang's study and our results.

There are some limitations to this study that should be noted. Firstly, each parameter of the PTFJ morphology was measured by a single investigator with an interval of 1 week between measurements. Increasing the number of investigators performing the measurements may increase the accuracy of the results. Secondly, the participants in the OA group and healthy group had a different age profile. It was difficult to obtain CT images from healthy subjects for this study because lower extremity CT scanning was not a conventional clinical test for healthy people in China, thus it was difficult to obtain CT images from healthy subjects in clinic. The data for the healthy group data was collected from a military physical examination, and thus the examined subjects were relatively younger than the OA patients. To eliminate the effects of age on our results, age was considered as a covariate in the regression model. The results from the regression model adjusted for age were consistent with those from the crude model. Thirdly, only K-L grades were included in this study to assess knee OA. The association between the PTFJ morphology and pain intensity and knee function in osteoarthritic knees may be considered in future studies. To further increase the reliability of the results, future studies may also consider using a larger sample size to further evaluate the relationship between the severity of knee OA and PTFJ morphology.

## Conclusions

This study found that the morphological characteristics of the PTFJ were significantly correlated with the presence of knee OA, particularly with regard to the inclination angle, declination angle and relative articular height. These findings advocated a potential way to screen for knee OA by assessing the morphology of the PTFJ. The results also indicated that altering the morphology of the PTFJ could potentially be used alleviate knee OA.

## Data Availability Statement

The raw data supporting the conclusions of this article will be made available by the authors, without undue reservation.

## Ethics Statement

The studies involving human participants were reviewed and approved by Beihang University Biological Science and Medical Engineering review board. The patients/participants provided their written informed consent to participate in this study.

## Author Contributions

X-ZQ and C-KC: conceptualization. X-ZQ and MZ: methodology and statistics analysis. X-ZQ, M-DN, and X-YM: software. MW, BZ, and X-HW: resources. X-ZQ: writing—original draft preparation. X-ZQ, MZ, H-ZW, and C-KC: writing—review and editing. C-KC: supervision. X-ZQ: takes responsibility for the integrity of the work as a whole, from inception to finished article. All authors have read and agreed to the published version of the manuscript.

## Conflict of Interest

X-HW is employed by the company Beijing Naton Technology Group Co. Ltd. The remaining authors declare that the research was conducted in the absence of any commercial or financial relationships that could be construed as a potential conflict of interest.
